# Metastatic colorectal cancer as the primary phenotype in a hereditary breast and ovarian cancer patient with Germline *BRCA1* mutation: a case report

**DOI:** 10.1186/s13048-022-01069-y

**Published:** 2022-12-03

**Authors:** Ying Liu, Jing Zhu, Xiao Wei, Duoxia Yang, Si Li, Xiaoping Qian, Li Li

**Affiliations:** 1 The Comprehensive Cancer Centre of Nanjing Drum Tower Hospital, The Affiliated Hospital of Nanjing University Medical School & Clinical Cancer Institute of Nanjing University, Nanjing, China; 2 Nanjing Drum Tower Hospital Clinical College of Nanjing Medical University, Nanjing, China; 3 Department of Pathology, Drum Tower Hospital, Nanjing University, Nanjing, China; 4 The State Key Lab of Translational Medicine and Innovative Drug Development, Jiangsu Simcere Diagnostics Co., Ltd, Nanjing, China

**Keywords:** Hereditary breast and ovarian cancer, Colorectal cancer, *BRCA1*, Diagnosis

## Abstract

Hereditary breast and ovarian cancer (HBOC) syndrome has increased predisposition to breast and/or ovarian cancer, and 24% of families with HBOC were associated with the germline pathogenic variants in *BRCA1/2*. Timely diagnosis and identification of mutation carriers is of utmost importance to improve survival benefit and quality of life. Cancers that have been included into screening of *BRCA1/2* associated HBOC included prostate and pancreatic cancers etc. In this case, we reported a patient who firstly presented symptoms of CRC and was finally diagnosed as *BRCA1* associated HBOC with advanced peritoneal carcinoma. With strategies of cetuximab based treatment and olaparib, and debulking surgeries, she has achieved an overall survival (OS) > 35 months. The aim was to indicate that HBOC might also first present as CRC, and comprehensive next-generation sequencing analysis might be a key complement for screening and diagnose of HBOC.

## Background

Hereditary breast and ovarian cancer (HBOC) syndrome is defined as an increased predisposition to breast and/or ovarian cancer [1], and 24% of families with HBOC were associated with the germline pathogenic variants in the BRCA1/2 [2–4]. Among patients diagnosed with HBOC, the risk-reducing surgery and targeted treatments have significantly improved survival benefit and quality of life [5, 6]. Therefore, timely diagnosis and identification of mutation carriers is of utmost importance in treatment of HBOC. Besides breast and ovarian cancer, HBOC was also reported to have an increased risk of other cancers such as prostate and pancreatic cancers etc. [7], which have been included into the testing criteria for HBOC susceptibility genes [8]. While whether colorectal cancer should be included into screening of *BRCA1/2* associated HBOC was not identified.

In this case, we reported a patient who first presented symptom of CRC and was finally diagnosed as *BRCA1* associated HBOC with advanced peritoneal carcinoma. With strategies of cetuximab based treatment and olaparib, and debulking surgeries, she has achieved an overall survival (OS) > 35 months. The aim was to indicate that HBOC might also first present as metastatic CRC, and comprehensive next-generation sequencing (NGS) analysis might be a key complement for screening and diagnose of HBOC or possible hereditary cancers.

## Case presentation

**Fig. 1:**
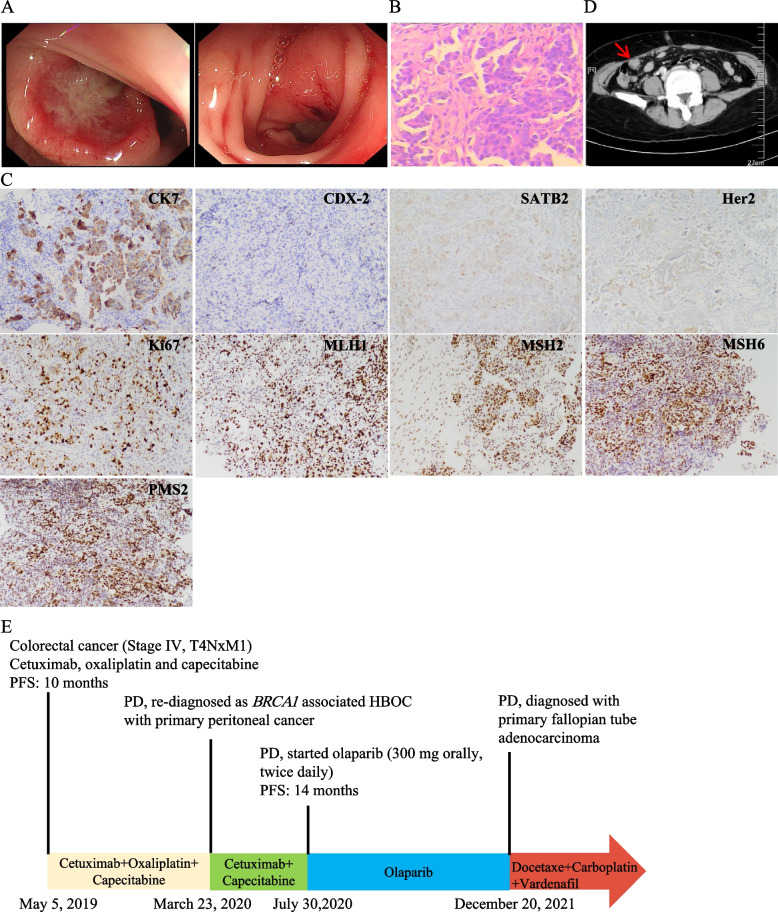
Imagological and pathological examination at first admission and timeline of diagnose and treatment. **A**. Left: Sigmoid colon lesion on colonoscopy; Right: Rectal lesion on colonoscopy; **B**. Hematoxylin-eosin (H&E) image, original magnification× 200; **C**. Immunohistochemical staining for CK7 (+++), CDX-2 (+), SATB2 (+), Her2 (+), Ki67 (50%+), MLH1 (+), MSH2 (+), MSH6 (+) and PMS2 (+) (original magnification× 100). 4-5 μm FFPE sections were used for IHC staining, antibodies used were listed in Table 1; **D**. Peritoneal lesion on CT (indicated by red arrow). **E**. Timeline of the patient’s diagnose and treatment, PFS is for progression-free survival

A 62-year-old female patient was referred to our hospital on May 5, 2019 with complaints of less and narrow stool with hematochezia for 3 months. Colonoscopy showed multiple protuberant ulcer lesions in both sigmoid colon and rectum (Fig. 1A), which was pathologically confirmed as poorly differentiated adenocarcinoma on Hematoxylin-eosin (H&E) (Fig. 1B). Immunohistochemistry (IHC) staining of tissues from colonoscopy were positive for CK7 (+++), CDX-2 (+), SATB2 (+), Her2 (+), Ki67 (50%+), MLH1 (+), MSH2 (+), MSH6 (+) and PMS2 (+) (Fig. 1C). The methods and materials of IHC were given in legends and Table 1. No lesions were found in breast and ovary at admissions except for peritoneal lesions (Fig. 1D). At the same time, CDX-2 (+) and SATB2 (+) was discussed as combination marker to discriminate colorectal carcinoma from other primary carcinoma [9, 10], and 19% of gastrointestinal adenocarcinoma with CK7 (+) /CDX-2 (+) /SATB2 (+) showed primary colorectal cancer [11]. Combined with the clinical manifestations, computed tomography (CT) scan, colonoscopy and pathological findings, the patient initially adopted treatment strategy for advanced colorectal cancer. The following targeted polymerase chain reaction (PCR) sequencing confirmed KRAS (−), NRAS (−), BRAF V600E (−). The patient subsequently received combination treatment of cetuximab, oxaliplatin and capecitabine (Fig. 1E). The disease showed partial response (near complete response).

**Table 1:** Antibodies used in this study

Antibody	Dilution	Pretreatment	Source
CK7	1: 200	EDTA9.0	Zhong Shan, Beijing, China
CDX-2	1: 200	EDTA9.0	Zhong Shan, Beijing, China
SATB2	Universal	EDTA9.0	Zhong Shan, Beijing, China
Her2	Universal	EDTA9.0	Roche Diagnostics, Basel, Switzerland
Ki67	1: 400	EDTA9.0	Zhong Shan, Beijing, China
MLH1	Universal	EDTA9.0	Dako Denmark A/S, Denmark
MSH2	Universal	EDTA9.0	Dako Denmark A/S, Denmark
MSH6	Universal	EDTA9.0	Dako Denmark A/S, Denmark
PMS2	Universal	EDTA9.0	Dako Denmark A/S, Denmark
Villin	Universal	EDTA9.0	Zhong Shan, Beijing, China
CK20	Universal	EDTA9.0	Zhong Shan, Beijing, China
ER	Universal	EDTA9.0	Roche Diagnostics, Basel, Switzerland
PAX8	Universal	EDTA9.0	Zhong Shan, Beijing, China
ERCC1	1: 200	EDTA9.0	Zhong Shan, Beijing, China
PR	Universal	EDTA9.0	Roche Diagnostics, Basel, Switzerland
P53	1: 200	EDTA9.0	Zhong Shan, Beijing, China
P16	1: 200	EDTA9.0	Zhong Shan, Beijing, China
WT1	1: 100	EDTA9.0	Mai Xin, Fuzhou, China
CD31	1: 200	EDTA9.0	Dako Denmark A/S, Denmark
D2-40	1: 200	EDTA9.0	Dako Denmark A/S, Denmark

**Fig. 2:**
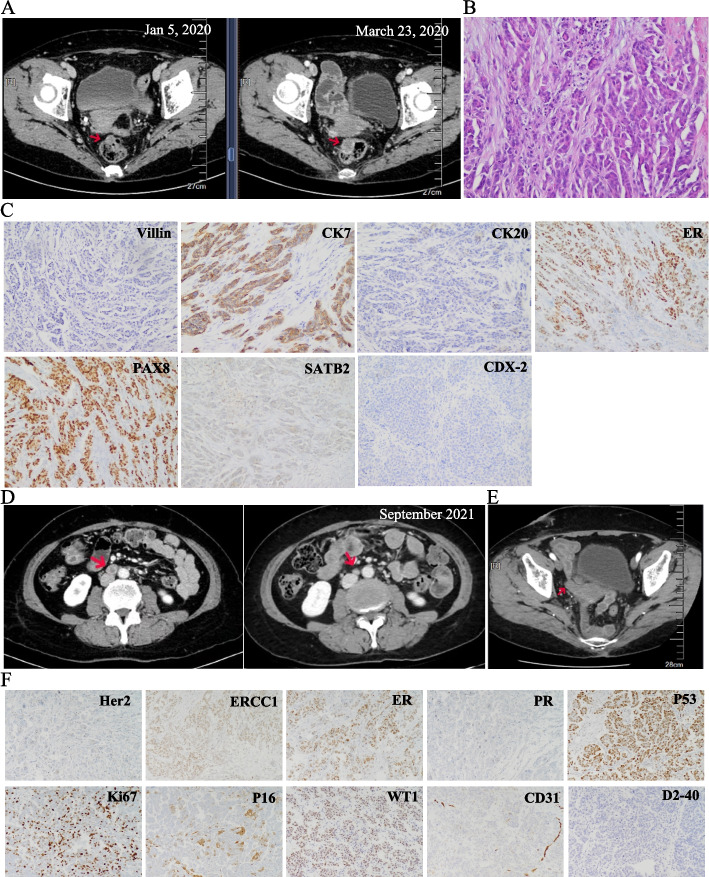
Re-diagnose of primary peritoneal cancer and progression on olaparib. **A**. Progression of rectal lesion after treatment of cetuximab, oxaliplatin and capecitabine for colorectal cancer, rectal lesion indicated by red arrow; **B**. Hematoxylin-eosin (H&E) staining pictures with the surgical sample. Original magnification× 200; **C**. Immunohistochemical staining results of Villin (−), CK7 (+++), CK20 (−), ER (++), PAX8 (+++), SATB2 (−) and CDX-2 (−) (original magnification× 100). Antibodies used were listed in Table 1; **D**. Left: CT scan showing enlarged abdominal lymph nodes (indicated by red arrow); Right: CT scan showing a partial response (PR) after 14 months of olaparib treatment; **E**. CT scan showing newly onset space occupying lesion (indicated by red arrow) in the right ovary. **F**. Immunohistochemical staining results of Her2(Br)-, ERCC1 (+), ER (+), PR (−), P53 (+), Ki67 (30%+), P16 (+), WT1 (+), CD31 (+), D2-40 (scattered+) (original magnification× 100). Antibodies used were listed in Table 1

While after about 10 months’ treatment, computed tomography (CT) scan revealed the enlargement of rectal lesion (Fig. 2A). The patient underwent a laparoscopic excision of rectal lesions on April 13, 2020. The second pathological examination was performed. Instead of arising from the muscular to serosal layer, which was the characteristic of primary colorectal cancer [12], the gross examination of surgical sample observed tumor tissues growing from the serosal layer, which indicate metastatic colorectal cancer (Fig. 2A&B). Further IHC staining revealed a status of Villin (−), CK7 (+++), CK20 (−), ER (++), PAX8 (+++), SATB2 (−) and CDX-2 (−) (Fig. 2C). Combined with results of the gross examination, IHC staining and imaging-observed lesions, she was further diagnosed as primary peritoneal carcinoma. To further clarify the molecular results, the surgical specimens were subjected to College of American Pathologists (CAP)-accredited laboratory for DNA-based NGS analysis (520 genes). The results showed that the patient harbored germline mutation in *BRCA1* (c.5470_5477delATTGGGCA, p.lle1824AspfsX3). Investigation of family history and NGS analysis (Table 2) showed that, the mother and sister were both diagnosed with breast cancer, and the father was diagnosed with prostate cancer. The cancer predisposition genes testing (28 gene-panel，College of American Pathologists (CAP)-accredited laboratory) was performed for the mother and brother. The same germline *BRCA1* mutation was detected with the mothers’ plasma sample. Our patient was finally diagnosed as HBOC, presenting peritoneal carcinoma with colorectal metastasis.

**Table 2:** Family history and gene mutations detected by NGS

Member	History of cancer	Germline mutation of ***BRCA1***	Concurrent mutation
Patient	Peritoneal carcinoma, fallopian tube adenocarcinoma, metastatic colorectal cancer	*BRCA1* (c.5470_5477delATTGGGCA, p.lle1824AspfsX3)	*Somatic mutation:* *TP53* (p. Tyr220Cys*)* *VHL* (p. Ser65Ala) *NF1* (exon41*-*46del) *FGF3* (p. Ala107Pro) *KMT2A* (p. Asp649Asn) *NBN* (p. Asp677Gly)
Mother	Breast cancer	*BRCA1* (c.5470_5477delATTGGGCA, p.lle1824AspfsX3)	Not performed
Father	Prostate cancer (Died)	–	–
Sister	Breast cancer (Died)	–	–
Brother	None	Wild-type	Not performed

The patient started olaparib treatment (300 mg orally, twice daily) on July 30, 2020. She then achieved partial response and a progression-free survival (PFS) of 14 months (Figs. 1E, 2D&E). During the maintenance treatment on olaparib after debulking surgery post-progression, a newly onset lesions in the right ovary was confirmed (Fig. 2E) on December 20, 2021. The patient received total abdominal hysterectomy bilateral salpingo oophorectomy with infracolic omentectomy. The IHC staining showed results of Her2 (Br)-, ERCC1 (+), ER (+), PR (−), P53 (+), Ki67 (30%+), P16 (+), WT1 (+), CD31 (+), D2-40 (scattered+) (Fig. 2F). A high-grade fallopian tube adenocarcinoma was thus considered. The patient then adopted combination of docetaxel and carboplatin and intraperitoneal injection of vardenafil since January 2022.

## Discussion

In this case we reported a patient with HBOC and presented initial symptom of metastatic colorectal cancer. HBOC was firstly brought up in 1866, when French physician Pierre P B reported a family with a familial predisposition to breast cancer. However, it was not until 1994 and 1995, *BRCA1* and *BRCA2*, DNA repair genes, were confirmed as the genetic defect to be associated with HBOC [2, 3]. In families with HBOC, the pathogenic mutations in *BRCA1* and *BRCA2* contributed to near 25% of HBOC cases in total [4]. Specially in women, *BRCA1* or *BRCA2* mutations result in a 57-65% or 45-55% risk of developing breast cancer by age 70 years, and a lifetime risk of 39-44% or 11-18% of developing ovarian cancer, respectively [1]. And that the phenotypes of HBOC in *BRCA1*/*BRCA2* mutation carriers are reported to have an age-dependent penetrance [5]. So, genetic testing for early diagnosis of HBOC is important.

Except for breast and ovarian cancer, patients with BRCA1/2 associated HBOC also showed higher risk of prostate cancer, uterine cancer, pancreatic cancer melanoma [7, 8]. In our case, although the patient was diagnosed as primary peritoneal carcinoma with colorectal metastasis, metastatic colorectal cancer was identified as the primary phenotype, which was barely reported. Due to the preliminary pathological examination with endoscopic specimens, no prior history of breast cancer and/or ovarian cancer in this patient and CRC was not formally included for HBOC screening, it is difficulty for accurate diagnosis in clinical practice. This case enlightened us that it might be helpful to perform further screening for BRCA1/2 associated HBOC among patients with CRC to fulfil early diagnose.

In this case, the gross examination on surgical samples of colorectal lesions gave us important leads on advanced CRC with peritoneal metastasis. We should also pay attention to IHC markers for CRC, since CK7(−) and CDX-2(+)/SATB2(+) were both reported to identify primary CRC with considerable accuracy [9, 13]. While, triple positive was observed during the patient’s first diagnose, however, primary CRC was finally excluded. The roles of CK7(−) and CDX-2(+)/SATB2(+) in diagnosing of CRC worth pondering. A comprehensive illustration on spectrum of primary cancer types in CK7(+)/CDX-2(+)/SATB2(+) tumors might assist in primary tumor identification as well.

Meanwhile, DNA-based NGS could be a key complement for screening and diagnose of HBOC, as well as other possible hereditary syndromes [14]. For instance, one of familial CRC, Lynch syndrome was associated with alterations in 4 DNA miss-match repair genes (*MSH2*, *MLH1*, *MSH6* and *PMS2*). A comprehensive analysis of germline and somatic gene alteration could help with the early diagnose and matching the individualized management approach. In our case, the same germline *BRCA1* was detected in our patient and observed in the mother with breast cancer. No concurrent germline alterations were detected. This provided important evidence of HBOC (Table 2). The patient then achieved a PFS of 14 months from olaparib. The first line cetuximab based treatment administrated under diagnose of CRC also brought a PFS of 10 months, it might also attribute to the anti-tumor effect of platin-based chemotherapy for ovarian cancer [15]. This indicated that the regimen might also be an adoptive therapy for this group of patients before conclusion of HBOC was drawn. Finally, the patient achieved an OS > 35 months before submission.

## Conclusion

In summary, our case presented a rare case of metastatic colorectal cancer as the primary phenotype of *BRCA1* associated HBOC and benefited from PARP (poly (ADP-ribose) polymerase) inhibitor of olaparib. Including susceptive CRC into screening criteria of *BRCA1* associated HBOC might be essential to give a precise management strategy.

## Data Availability

The data presented in this study are available on request from the corresponding author. The data are not publicly available due to restrictions of patient privacy.

## References

[1] Nielsen FC, van Overeem HT, Sørensen CS (2016). Hereditary breast and ovarian cancer: new genes in confined pathways. Nat Rev Cancer.

[2] Miki Y, Swensen J, Shattuck-Eidens D, Futreal PA, Harshman K, Tavtigian S (1994). A strong candidate for the breast and ovarian Cancer susceptibility gene *BRCA1*. Science..

[3] Wooster R, Bignell G, Lancaster J, Swift S, Seal S, Mangion J (1995). Identification of the breast cancer susceptibility gene BRCA2. Nature..

[4] Kast K, Rhiem K, Wappenschmidt B, Hahnen E, Hauke J, Bluemcke B (2016). Prevalence of *BRCA1/2* germline mutations in 21 401 families with breast and ovarian cancer. J Med Genet.

[5] Hartmann LC, Lindor NM (2016). The role of risk-reducing surgery in hereditary breast and ovarian Cancer. Longo DL, editor. N Engl J Med.

[6] Pujade-Lauraine E, Ledermann JA, Selle F, Gebski V, Penson RT, Oza AM (2017). Olaparib tablets as maintenance therapy in patients with platinum-sensitive, relapsed ovarian cancer and a BRCA1/2 mutation (SOLO2/ENGOT-Ov21): a double-blind, randomised, placebo-controlled, phase 3 trial. Lancet Oncol.

[7] Yoshida R (2021). Hereditary breast and ovarian cancer (HBOC): review of its molecular characteristics, screening, treatment, and prognosis. Breast Cancer.

[8] Daly MB, Pal T, Berry MP, Buys SS, Dickson P, Domchek SM (2021). Genetic/familial high-risk assessment: breast, ovarian, and pancreatic, version 2.2021, NCCN clinical practice guidelines in oncology. J Natl Compr Cancer Netw.

[9] Dabir PD, Svanholm H, Christiansen JJ (2018). SATB2 is a supplementary immunohistochemical marker to CDX2 in the diagnosis of colorectal carcinoma metastasis in an unknown primary. APMIS..

[10] Berg KB, Schaeffer DF (2017). SATB2 as an Immunohistochemical marker for colorectal adenocarcinoma: a concise review of benefits and pitfalls. Arch Pathol Lab Med.

[11] Chauhan A, Sanchez-Avila M, Manivel J, Dachel S, Larson W, Hanson B (2021). Optimization of Immunophenotypic panel to differentiate upper from lower gastrointestinal adenocarcinomas: analysis of new and traditional markers. Appl Immunohistochem Mol Morphol.

[12] Weiser MR (2018). AJCC 8th edition: colorectal Cancer. Ann Surg Oncol.

[13] Meagher NS, Wang L, Rambau PF, Intermaggio MP, Huntsman DG, Wilkens LR (2019). A combination of the immunohistochemical markers CK7 and SATB2 is highly sensitive and specific for distinguishing primary ovarian mucinous tumors from colorectal and appendiceal metastases. Mod Pathol.

[14] Ma H, Brosens LAA, Offerhaus GJA, Giardiello FM, de Leng WWJ, Montgomery EA (2018). Pathology and genetics of hereditary colorectal cancer. Pathology..

[15] BRCA mutation frequency and patterns of treatment response in BRCA mutation-positive women with ovarian cancer: a report from the Australian Ovarian Cancer Study Group - PubMed [Internet]. Available from: https://pubmed.ncbi.nlm.nih.gov/22711857/. [Cited 2022 Jun 9]. 10.1200/JCO.2011.39.8545 PMC3413277 22711857

